# Increased prosocial value orientation in autistic adults

**DOI:** 10.1177/13623613251385029

**Published:** 2025-10-28

**Authors:** Paul AG Forbes, Gillian Hughes, Leonhard Schilbach, Sarah White, Tobias Kalenscher

**Affiliations:** 1 Heinrich-Heine University Düsseldorf, Germany; 2 University College London, UK; 3 Heinrich Heine University Düsseldorf, Germany; 4 Ludwig-Maximilians-Universität, Germany

**Keywords:** fairness, generosity, prosocial behaviour, social cognition, social decision-making

## Abstract

Social discounting describes the tendency to give fewer resources to those we feel less close to. Previous work suggests autistic individuals show a flatter decline in generosity towards socially distant others compared with non-autistic participants. We investigated whether this enhanced prosociality towards socially distant others in autism was driven by genuinely higher prosociality or instead a preference for repetitive responding. We measured prosocial preferences in 37 autistic and 38 non-autistic adults using the social value orientation questionnaire, where participants allocated money between themselves and people at six different social distances (e.g. friend vs stranger). We replicated previous findings by showing that autistic adults were more prosocial than non-autistic adults towards more socially distant others. Crucially, these effects were not driven by more repetitive responding in autism, nor by differences in attitudes to money. While autistic people often face challenges navigating their social worlds, our findings reinforce the view that autism is also associated with more prosociality. We argue that differences in fairness norms could drive more prosociality in autism, but this remains to be tested in future work.

**Lay abstract:**

Sharing and giving to others are important for our social relationships. Previous studies show that when given opportunities to share money, autistic and non-autistic people give the same amount of money to people they feel close to, like their friends. However, compared with non-autistic people, autistic people give more money to people they feel less close to, like strangers. In this study, we replicated this finding. Compared with non-autistic participants, autistic participants were more generous to people they did not feel close to. We also found that this increased generosity in autism was not the result of autistic participants responding more repetitively in the task. Autistic and non-autistic participants also showed similar attitudes towards money. We propose that some autistic people could be more generous because they show differences in how they think about fairness. But future studies will need to look at this more closely. We hope that our results can help to change the way people think about social behaviour in autism. While autistic people often face challenges navigating their social worlds, autism can also be associated with more generosity.

## Introduction

Many social decisions involve sharing resources with others. Social discounting refers to the tendency to share fewer resources with people at greater social distances – those we feel less close to (Jones & Rachlin, 2006). Autism is characterised by differences in social behaviour (American Psychiatric Association, 2013), and three previous studies have investigated social discounting in autism. Two studies (Forbes et al., 2024; Tei et al., 2019) showed that autistic adults were as generous as non-autistic participants when sharing resources with close others (e.g. friends) but were *more* generous towards more socially distant others (e.g. strangers). Declines in generosity at increasing social distance were, therefore, less steep in autism. Conversely, Warnell et al. (2019) showed that autistic adolescents and young adults were less generous to close others compared with a non-autistic group in a social discounting task. Differences in task design and age could have driven differences between studies. Thus, we used a novel approach to investigate social discounting in autism. We examined whether differences in social discounting were due to genuine increases in prosociality in autism or due to a preference for repetitive responding, as explained below.

Both studies showing enhanced generosity in autism used the same social discounting task in which participants chose between two options on each trial (Sellitto et al., 2021). The more generous option was fixed and the same on every trial: an equal split of the money between oneself and another person. The less generous option varied across trials. As autism is associated with an insistence on sameness and repetitive behaviour (American Psychiatric Association, 2013), it is possible that increased generosity in autism was simply a byproduct of autistic participants’ tendency to select the same option on each trial, that is, a preference for repetitive responding rather than prosociality.

**Figure 1.**
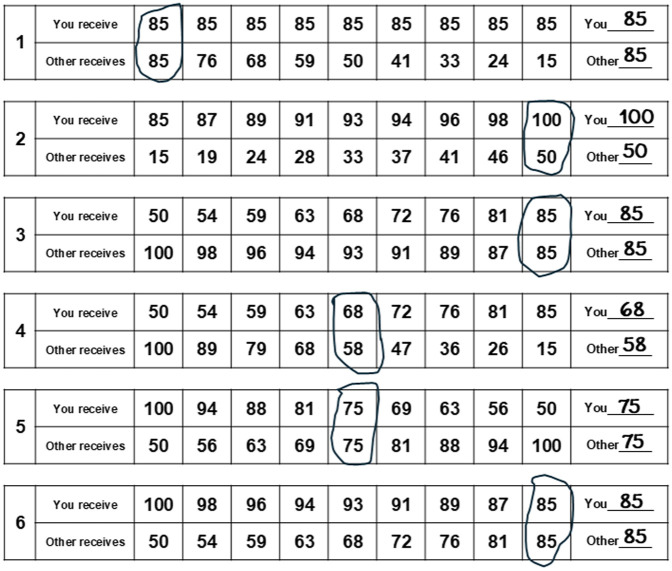
The responses of a fictional participant in the six-item social value orientation questionnaire (Murphy et al., 2011), where participants allocated money between themselves and another person. The SVO angle is calculated by taking the mean allocated to self across the six items (83 in the above example, i.e. the mean of 85, 100, 85, 68, 75, 85) and subtracting 50 (83 − 50 = 33) and then doing the same for allocations to the other (73 − 50 = 23). The ratio between the mean allocation to others and the mean allocation to self is calculated (23/33 = 0.697). This ratio is converted into an angle using the inverse tangent (0.609 radians) and converted to degrees for interpretability (0.609 × 180/π = 34.88°). A larger positive angle indicates a greater relative allocation to others compared with self and indicates a more prosocial orientation. Participants completed the SVO questionnaire six times: once for each person at each social distance (for the full questionnaire, see Supplemental Appendix 1).

To test this, we used a well-established tool to measure prosocial preferences: the six primary items of the social value orientation (SVO) questionnaire (Murphy et al., 2011). Participants chose between distributions of money between themselves and another person on a nine-point slider (Figure 1). Crucially, each of the six items was unique, so it was not possible to repeatedly choose the same distribution on each item. Choosing the same relative position on the slider on each trial (e.g. the leftmost option) would result in different distributions between oneself and another person. By combining allocations to self and other across the six items, an SVO angle could be calculated, providing a continuous measure of participants’ prosocial tendencies. A larger angle indicated greater prosociality. To determine whether the SVO angle differed across social distances, participants completed the SVO questionnaire for people at six social distances, ranging from someone emotionally close to a complete stranger.

We predicted an interaction between group (non-autistic, autistic) and social distance. Specifically, we predicted that the flatter decline in SVO angle at increasing social distance in autism would be driven by increased prosociality to more socially distant others (Forbes et al., 2024; Tei et al., 2019). Second, we aimed to determine whether increased prosociality was due to more repetitive responding in autism: the tendency to select the same response. Finally, participants completed a questionnaire to determine whether differences in attitudes towards money drove differences in prosociality in autism (Furnham et al., 2012).

## Method

### Participants

In total, 37 autistic and 38 non-autistic participants took part (see Supplemental Materials for power calculations), significantly exceeding the sample sizes from previous studies (Forbes et al., 2024; Tei et al., 2019). Participants were recruited via a local database of participants at University College London and came to the lab as part of a research day in which they took part in several studies. The groups were comparable on age, gender and IQ scores but showed large differences in autistic traits (see Table 1). All autistic participants had a formal diagnosis of autism from an independent clinician.

**Table 1.** Details of the autistic and non-autistic participants who were matched on age, gender and IQ.

	Non-autistic	Autistic	*P*-value
Age (*N* = 37:37)	39.32 (12.59)	34.46 (10.65)	0.077
Gender	16M; 22 F	23M; 14 F	0.082
AQ^a^ (*N* = 38:35)	16.63 (7.55)	33.86 (8.51)	<0.001
Verbal IQ^b^ (*N* = 37:36)	107.22 (10.75)	111.17 (13.91)	0.180
Non-verbal IQ^b^ (*N* = 37:36)	104.41 (14.66)	110.22 (16.29)	0.113
Full-scale IQ^b^ (*N* = 37:36)	107.24 (12.48)	112.00 (15.30)	0.151

a Autism Quotient (Baron-Cohen et al., 2001).

b Verbal and non-verbal IQ were measured using the Wechsler Adult Intelligence Scale-Fourth Edition (Wechsler, 2012) or Wechsler Abbreviated Scale of Intelligence (Wechsler, 1999).

### Measures

Participants completed the six primary items of the SVO questionnaire (Murphy et al., 2011): participants were required to distribute money between themselves and another person (for a detailed explanation, see Figure 1; for the full questionnaire, see Supplemental Appendix 1). Based on allocations to self and other, an SVO angle could be calculated (ryanomurphy.com/styled-2/styled-4/), ranging from competitive (<−12.0°), individualistic (from −12.04° to 22.45°), and prosocial (from 22.45° to 57.15°) to altruistic (>57.15°). Higher angle values correspond to a stronger prosocial orientation. Participants completed the SVO questionnaire six times for people at different social distances.

The concept of social distance was explained as in previous studies (Forbes et al., 2024). Participants were told that social distance refers to how emotionally close they feel to someone. So, someone at social distance 1 is the person who is most important to them and to whom they are emotionally closest. To help visualise social distance, participants were presented with a 100-point line showing themselves as a purple figure on the left side and then a yellow figure representing another person positioned along this line at different social distances. Participants were asked to think of a specific person at social distances 1, 5, 10 and 20 to write down their relationship to that person and the person’s initials. Participants were told not to think of anyone with whom they share a household or bank account, nor anyone they have negative feelings towards. When participants completed the SVO items for each person, they were asked to write down the initials of this person again, so they had that person in mind when making their decisions. For social distance 50, participants were told that this was someone they had met before but whose name they could not remember, and social distance 100 was a complete stranger.

To ensure participants understood the task, they were asked four multiple-choice questions. Decisions were incentivised: participants were informed that across all participants, a computer would randomly select 12 choices, which would be paid out. This ensured that the decisions could lead to actual financial gains. There were four versions of the questionnaire to counterbalance the order in which the social distances (SD) appeared:

1. SD50, SD1, SD20, SD5, SD100, SD10
2. SD10, SD100, SD5, SD20, SD1, SD50
3. SD20, SD1, SD50, SD10, SD100, SD5
4. SD5, SD100, SD10, SD50, SD1, SD20

Finally, participants indicated the extent to which they agreed with 16 statements (1 = strongly disagree; 5 = strongly agree) relating to money attitudes in terms of power (e.g. ‘Money is important because it shows how successful and powerful you are’), freedom ( ‘There are very few things money can’t buy’), love ( ‘I am very generous with the people I love’) and security ( ‘I rather save money than spend’) (Furnham et al., 2012).

All participants provided written informed consent, and the study was approved by the University College London Institute of Cognitive Neuroscience Local Research Ethics Committee (project number 2025-0086-305). Data and code are available on the Open Science Framework (https://osf.io/h6z3f/?view_only = be18df30172149739cae4747b72590c9)

## Results

### Increased prosocial value orientation to socially distant others in autism

For each participant, we calculated one SVO angle for each of the six social distances. For one autistic participant, one SVO angle was missing (social distance 1), as they entered a distribution which was not available on the slider. A linear mixed effects model was performed using the *lme4* function in R (Bates et al., 2015). This determined whether the effect of social distance was dependent on group, that is, if the decline in SVO angle with increasing social distance would be steeper in the non-autistic group versus the autistic group. We included the interaction between two predictors in the model: ‘group’ (autistic vs non-autistic) and ‘rank social distance’. Social distance was linearised (rank social distance) as the gaps between the social distances were not incremental (i.e. 1, 5, 10, 20, 50, 100) (Forbes et al., 2024). We included random intercepts for participants and random slopes for the within-subject factor ‘rank social distance’ (Barr et al., 2013; Matuschek et al., 2017).

**Figure 2.**
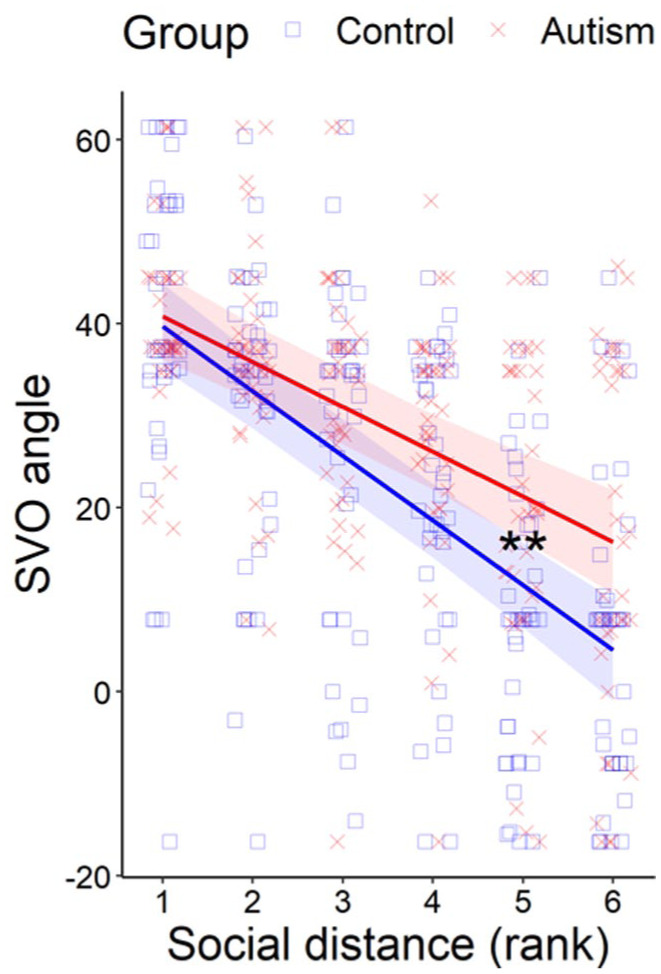
SVO angle (higher values indicate greater prosociality) in the autistic and non-autistic control group at each social distance. Individual data points for each participant are shown with red crosses and blue squares. Solid lines and ribbons are the model estimates for the interaction effects between group and rank social distance, plus 95% confidence intervals. ** *p* < 0.01.

We found a significant interaction between rank social distance and group (estimate = −2.14, *SE* = 0.980, *p* = 0.032). Simple slopes analysis revealed a steeper social discounting slope in the non-autistic group (estimate = −7.03, *SE* = 0.688, *p* < 0.001) compared with the autistic group (estimate = −4.89, *SE* = 0.698, *p* < 0.001; Figure 2). Post-hoc *t*-tests (Table 2) revealed a significant difference at social distance 50 (Bonferroni-corrected) as well as social distance 20 and 100 (uncorrected), with the autistic group showing more prosocial SVO angles than the non-autistic group.

**Table 2.** Group differences in SVO angle at different social distances.

	Mean SVO angle (*SD*)			
Social distance (rank)	Non-autistic *n* = 38	Autistic *n* = 37	*P*-value Bonferroni-corrected	Cohen’s *d*
1 (1)	40.45 (17.94)	39.62 (10.31)	1.00	0.056
5 (2)	30.57 (15.81)	35.72 (12.23)	0.707	−0.364
10 (3)	26.68 (18.25)	31.90 (13.88)	1.00	−0.322
20 (4)	21.29 (16.40)	28.17 (13.24)	0.296	−0.461
50 (5)	7.79 (15.51)	20.42 (16.71)	0.007**	−0.783
100 (6)	5.95 (16.82)	15.33 (19.11)	0.163	−0.522

Note, group differences were present at SD50 after correction, but at SD20 (*p* = 0.049), SD50 (*p* < 0.001) and SD100 (*p* = 0.027) before correction.

** < 0.01.

### No group differences in repetitive responding or money attitudes in autism

To check whether the effect was driven by autistic participants responding more repetitively across social distances, we calculated the mean number of unique SVO values across the six social distances. There were no differences (*p* = 0.701) between the autistic (*M* = 4.54, *SD* = 1.65) and non-autistic group (*M* = 4.68, *SD* = 1.56). The number of participants who had the same SVO angle across all social distances was the same in both groups (*n* = 3 per group). Thus, the greater prosociality at increasing social distance in autism was not driven by a tendency to make the same response at every social distance. Finally, there were no significant group differences in money attitudes on any subscales (all *p*s > 0.38; see Supplemental Table S1).

## Discussion

Autistic participants were more prosocial towards socially distant others than non-autistic participants. There were no differences in prosociality towards close others between the groups. This replicates two previous studies (Forbes et al., 2024; Tei et al., 2019) and extends these findings by demonstrating that a more prosocial orientation in autism was not due to participants simply responding more repetitively. The number of unique SVO angles across social distances was not different between the autistic and non-autistic groups. In addition, there were no differences in attitudes to money in autism, supporting previous findings (Cage et al., 2013). Thus, three independent samples from three different countries (Japan, Germany, and the UK) have converged on the finding that autistic adults show enhanced prosociality to more socially distant others.

We propose that a more consistent implementation of fairness norms in autism could drive the effects (Forbes et al., 2024; Ikuse et al., 2018; Klapwijk et al., 2017). This is supported by the finding that autistic individuals make more consistent decisions (Farmer et al., 2017), are more inflexible when following moral rules (Hu et al., 2021), and are more likely to endorse fairness as a foundational principle for their moral outlook (Greenberg et al., 2024). An aim for future work will be to examine fairness more precisely in autism. Here, the secondary items of the SVO questionnaire could help distinguish whether a prosocial motive in autism is driven by inequality aversion or a motivation to maximise joint outcomes (Murphy et al., 2011). Moreover, understanding how fairness develops in autism is especially important (Ryan-Enright et al., 2022), given that one study found that autistic adolescents and young people were *less* generous to close others in a social discounting task (Warnell et al., 2019). Finally, all studies on social discounting in autism have focused exclusively on autistic individuals in high-income countries with lower support needs and the capacity for verbal speech. Future work will need to test the generalisability of these findings in the broader autistic community.

To conclude, autistic individuals showed enhanced prosocial behaviour, replicating previous work. Compared with non-autistic participants, autistic adults were more generous towards people they felt less close to. We extend previous work by showing that these effects were not due to more repetitive responding in autism nor due to differences in attitudes towards money. Our findings support an emerging view that while autistic people often face challenges navigating their social worlds (Hull et al., 2017), autism is associated with more prosocial behaviour (Forbes et al., 2024). Understanding whether greater fairness in autism drives this prosociality should be an aim for future work.

## Supplemental Material

sj-pdf-1-aut-10.1177_13623613251385029 – Supplemental material for Increased prosocial value orientation in autistic adults Supplemental material, sj-pdf-1-aut-10.1177_13623613251385029 for Increased prosocial value orientation in autistic adults by Paul AG Forbes, Gillian Hughes, Leonhard Schilbach, Sarah White and Tobias Kalenscher in Autism
