# Supplementary material for: Increased prosocial value orientation in autistic adults
Source: Autism. 2025 Oct 28;30(2):538–43. doi: 10.1177/13623613251385029 (PMC12804402; doi:10.1177/13623613251385029)
Supplement: sj-pdf-1-aut-10.1177_13623613251385029 – Supplemental material for Increased prosocial value orientation in autistic adults [file sj-pdf-1-aut-10.1177_13623613251385029.pdf]

Participant code: ______________________ **Date:** ______________________

In this task you will be making a series of decisions about allocating resources between you and other people. For each question, please indicate the distribution you prefer most by marking the respective position along the midline. You can only make one mark for each question.

Your decisions will yield money for both your and another person. In the example below, a person has chosen to distribute money so that they receive 50 pence, while the other person receives 40 pence.

There are no right or wrong answers, this is all about personal preferences. After you have made your decision, **write the resulting distribution of money on the spaces on the right**. As you can see, your choices will influence both the amount of money you receive as well as the amount of money the other person receives.

Example:

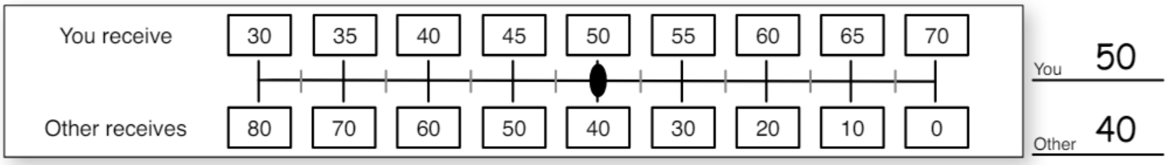

The other person you will be sharing money will vary. In each round, we will tell you with whom you can share money by using a picture. The picture looks something like this:

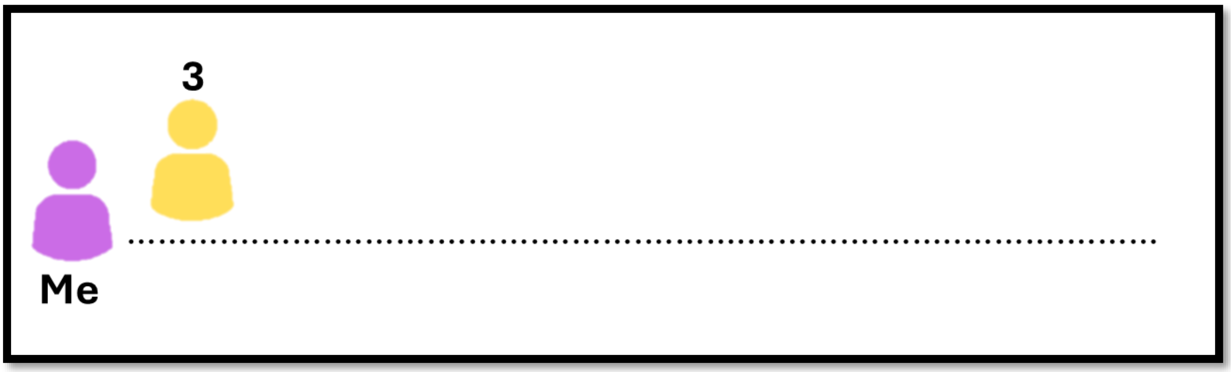

In this picture, you see a purple figure on the left, this is you. You also see a yellow figure - this is the other person you can share money with.

If the yellow figure is very close to you (the purple figure), we ask you to think of a person you care about very much emotionally. The further away the yellow figure is from you (the purple figure) the less you care about this person emotionally.

The number above the yellow figure helps you to tell how far away it is from you, that is, how much or how little you care about this person emotionally. The number goes from 1 (the person you care most about) to 100 (a person you do not care about at all). We will give you some examples now to explain this.

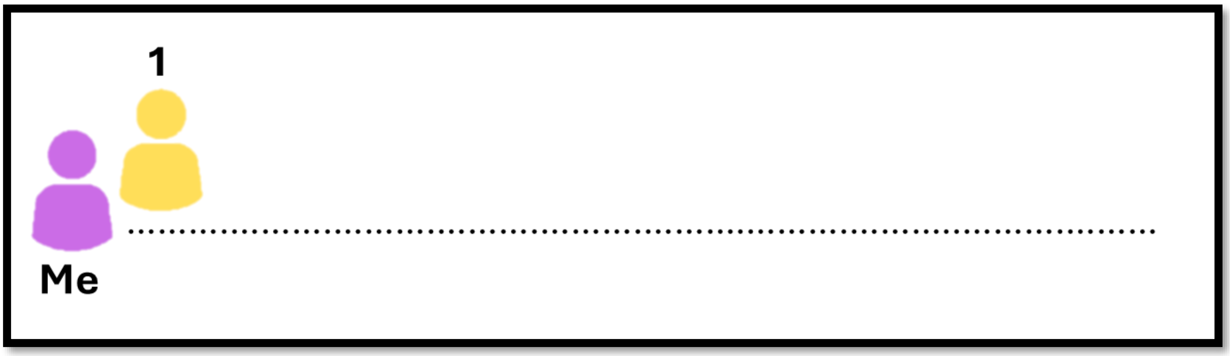

In this picture, the yellow figure has the **number 1**. This is a person who is most important to you and to whom you are emotionally closest. This could be, for example, your best friend.

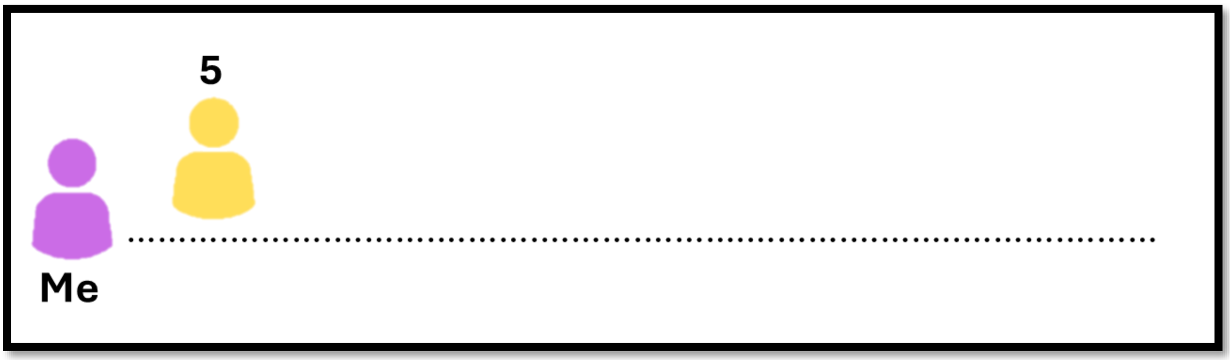

In this picture, the yellow figure with the **number 5** is a person who is important to you. You still feel emotionally close to this person but not as close as to the person at position 1.

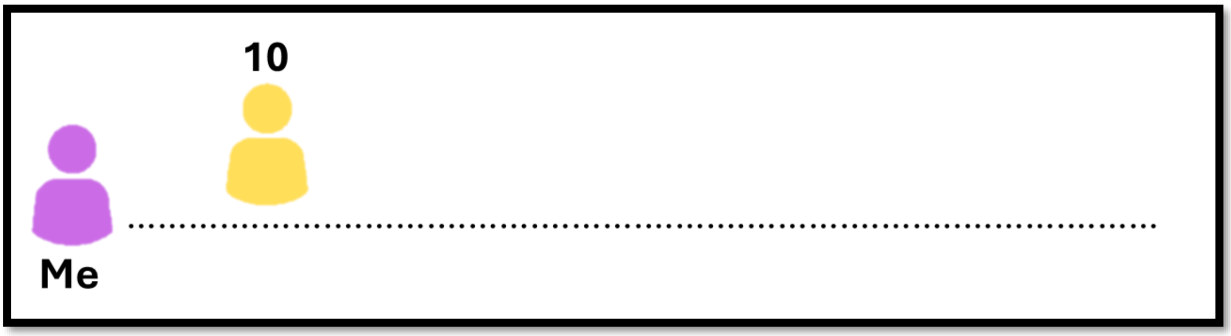

In this picture, the yellow figure has the **number 10** and is a person you feel less emotionally close to than the people before but still reasonably close.

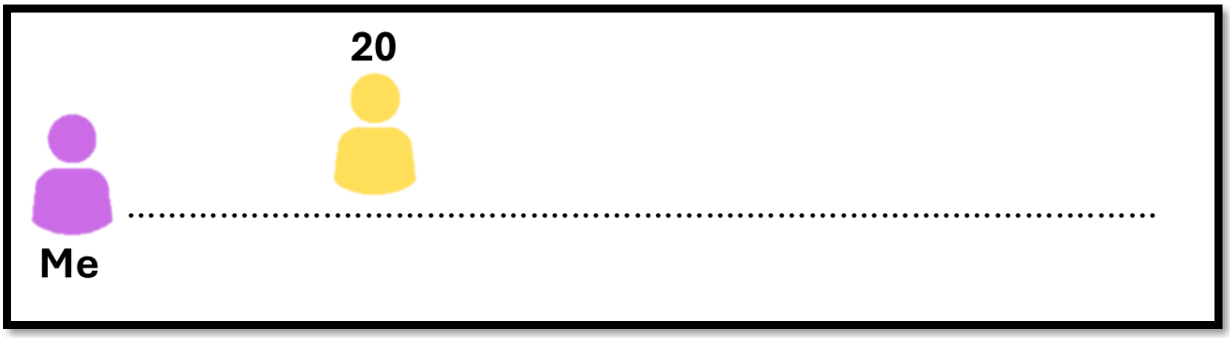

In this picture, the yellow figure has the number 20 and is a person you feel even less emotionally close to than the people before but still someone you feel some closeness towards.

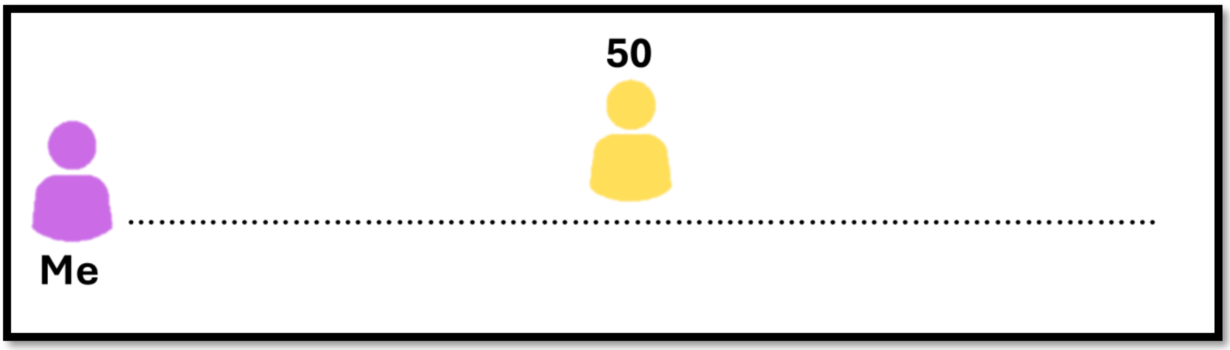

In this picture, the yellow figure has the number 50 and is a person you have seen a few times, but whose name you do not know.

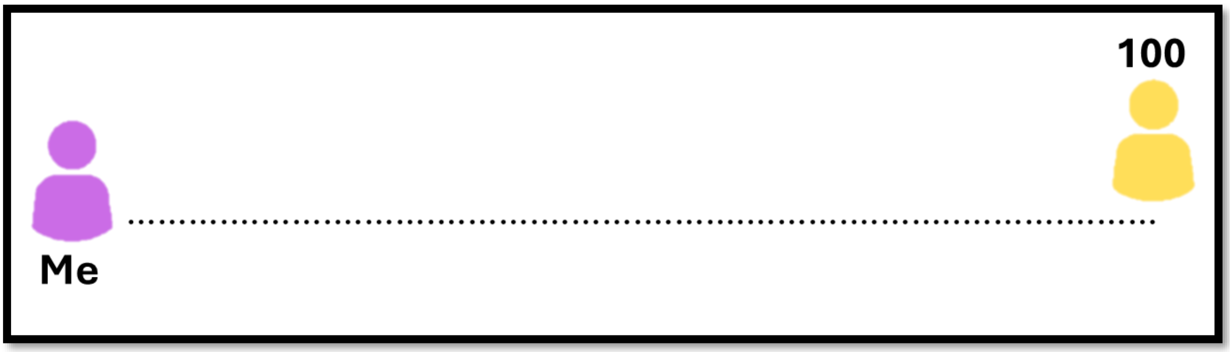

In this picture, the yellow figure has the number 100 and is a stranger who you have never seen before. But this would be someone you have no negative feelings towards.

Next, we will show you the pictures again. We will ask you to tell us who you think of when you see these pictures.

Very important! Please always think of a person whom you have no negative feelings towards. And **please do not think of a person if they live under the same roof as you or if you share a bank account with them**. For example, do not think of your partner, your siblings, your parents, or children if they live under the same roof as you or if you share a bank account with them. The reason is that money you would share with such a person would also benefit you.

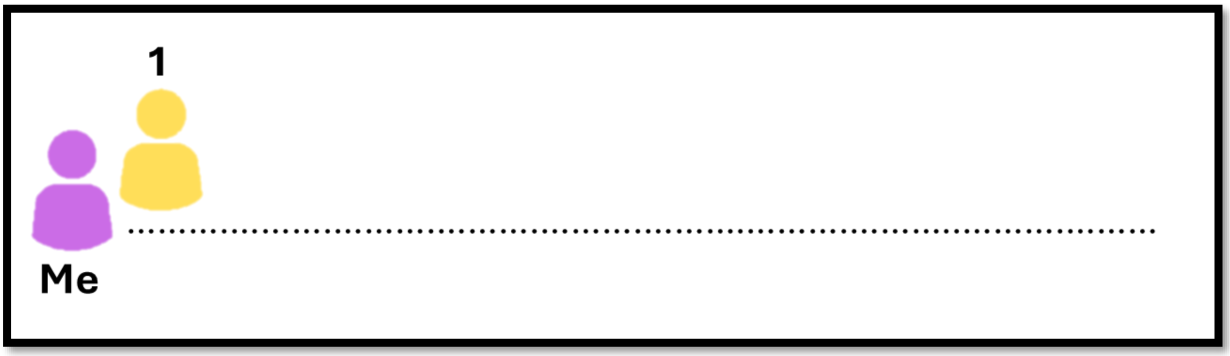

In this picture, the yellow figure has the **number 1**. This is a person who is most important to you and to whom you are emotionally closest. This could be, for example, your best friend. But please do not think of a person if they live under the same roof as you or if you share a bank account with them.

Who do you think of when you see this picture? Please write their initials and your relationship to them (e.g. for Jo Smith my best friend, I would write **"JS, best friend")**

Initials: ____________________ **Relationship with this person:** ____________________

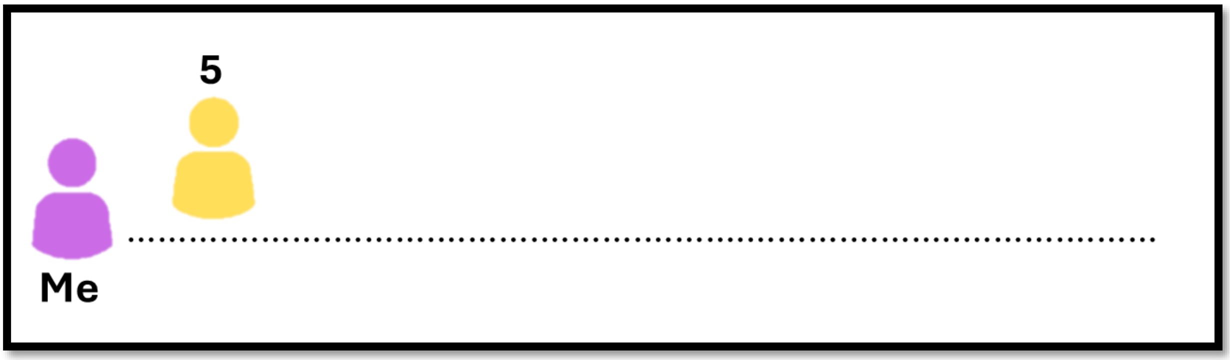

In this picture, the yellow figure has the **number 5** and is a person who is important to you. You still feel emotionally close to this person but not as close as the person at position 1.

Who do you think of when you see this picture? Please write their initials and your relationship to them.

Initials: ____________________ **Relationship with this person:** ____________________

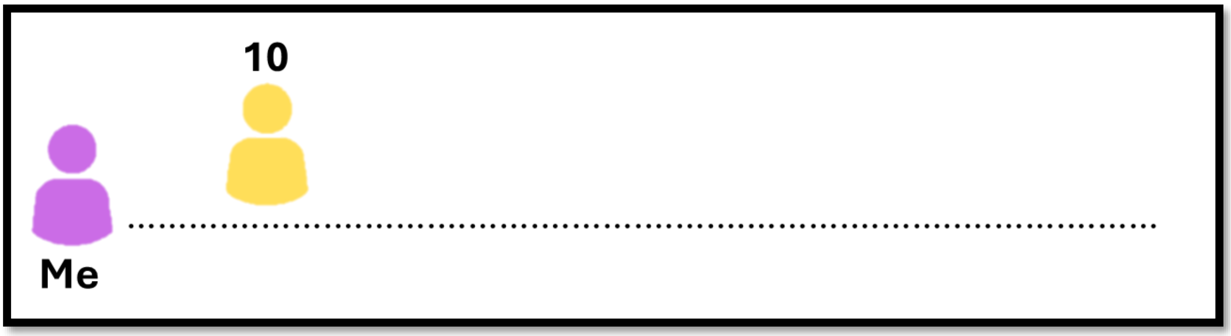

In this picture, the yellow figure has the **number 10** and is a person you feel less emotionally close to than to the other people before but still reasonably close.

Who do you think of when you see this picture? Please write their initials and your relationship to them.

Initials: ____________________ **Relationship with this person:** ____________________

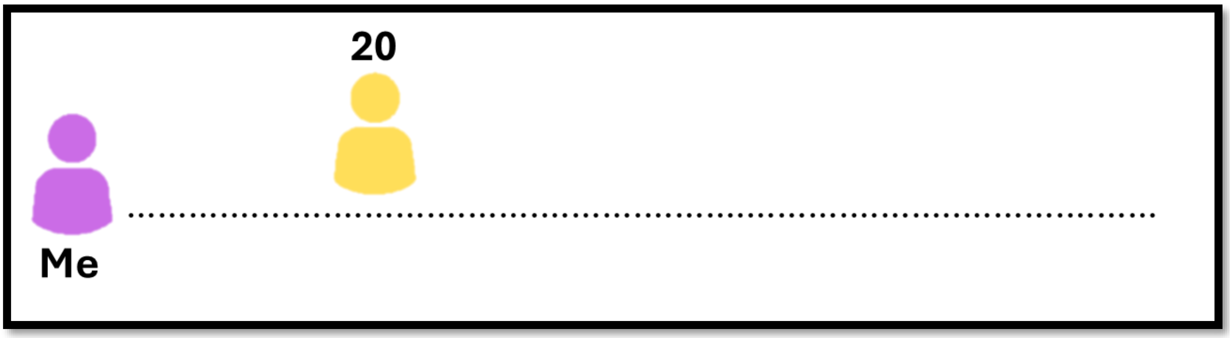

In this picture, the yellow figure has the number 20 and is a person you feel even less emotionally close to than to the other persons before but still someone you feel some closeness towards.

Who do you think of when you see this picture? Please write their initials and your relationship to them.

Initials: ____________________ **Relationship with this person:** ____________________

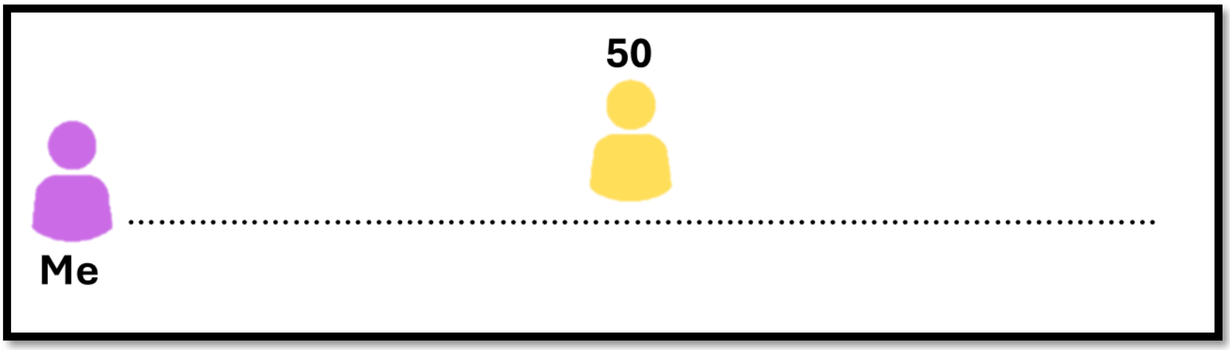

As a reminder in this picture, the yellow figure has the number 50 and is a person you have seen a few times, but whose name you do not know. You cannot write down anyone for this picture.

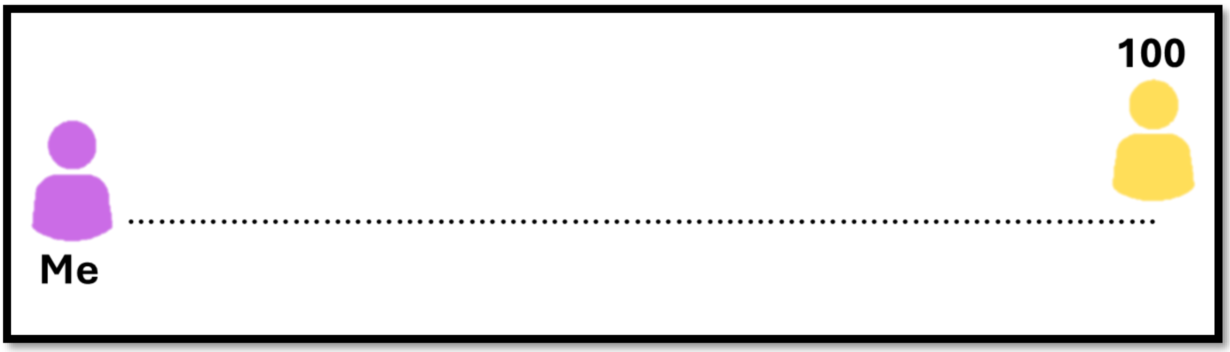

As a reminder in this picture, the yellow figure has the number 100 and is a stranger who you have never seen before. You cannot write down anyone for this picture.

You will shortly complete the following **six questions** for the people at the different positions. Each arrow pointing to the right indicates one question. For each of the six questions, please indicate the distribution you prefer most by marking the respective position along the midline. You can only make one mark for each question. After you have made your decision, write the resulting distribution of money on the spaces on the right as in the example.

Example:

You receive	30	35	40	45	50	55	60	65	70	You	50
Other receives	80	70	60	50	40	30	20	10	0	Other	40

The six questions:

1

You receive	85	85	85	85	85	85	85	85	85	You	
Other receives	85	76	68	59	50	41	33	24	15	Other	

2

You receive	85	87	89	91	93	94	96	98	100	You	
Other receives	15	19	24	28	33	37	41	46	50	Other	

3

You receive	50	54	59	63	68	72	76	81	85	You	
Other receives	100	98	96	94	93	91	89	87	85	Other	

4

You receive	50	54	59	63	68	72	76	81	85	You	
Other receives	100	89	79	68	58	47	36	26	15	Other	

5

You receive	100	94	88	81	75	69	63	56	50	You	
Other receives	50	56	63	69	75	81	88	94	100	Other	

6

You receive	100	98	96	94	93	91	89	87	85	You	
Other receives	50	54	59	63	68	72	76	81	85	Other	

Very important! At the end of this year's autism@ICN data collection period, across the entire study and all participants, a computer will randomly select 12 choices.

If your choice is chosen, we will see how much money you wanted to keep for yourself, and how much money you wanted to share with another person **across the six questions for that person**. In other words, we will add up the amounts you wanted to keep for yourself and the amounts you wanted to share with the other person across the six questions.

Depending on how you decide to allocate the money, this could lead to about £5 for yourself and £5 for another person. If your choice is chosen, we will contact you to give you the money that you decided to keep for yourself. The money you decided to share with the person will be given to that person.

To make sure you understand the rules of the task, we're going to ask you some questions. Just circle the answer you think is correct. If you are unsure, please discuss the questions with the experimenter.

Each decision…

…involves another person and me.

…involves only me.

…involves only another person.

The yellow figure on the right side of the picture…

…is irrelevant for the decisions.

…represents me.

…represents another person I can share money with.

The purple figure on the left side of the picture…

…is irrelevant for the decisions.

…represents me.

…represents another person I can share money with.

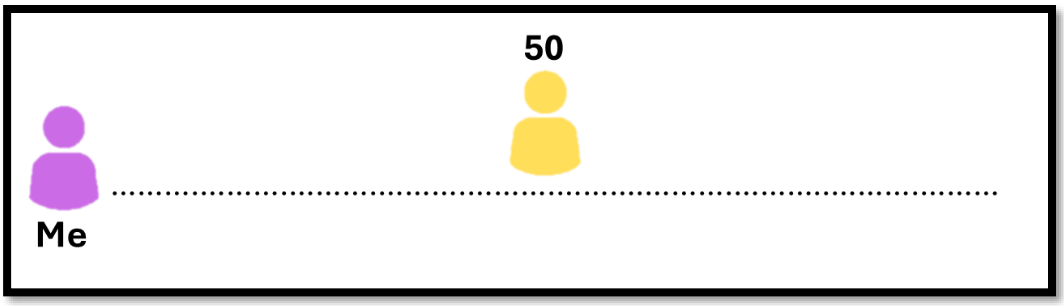

In this round you will be making a series of decisions about allocating resources between you and the person you chose for **position 50**.

This is a person you have seen a few times, but whose name you do not know.

Each arrow pointing to the right indicates one question. For each question, please indicate the distribution you prefer most by marking the respective position along the midline. You can only make one mark for each question.

Your decisions will yield money for both you and the person at **position 50**. There are no right or wrong answers, this is all about personal preferences. After you have made your decision, write the resulting distribution of money on the spaces on the right.

1

You receive	85	85	85	85	85	85	85	85	85
Other receives	85	76	68	59	50	41	33	24	15

You ________
Other ________

2

You receive	85	87	89	91	93	94	96	98	100
Other receives	15	19	24	28	33	37	41	46	50

You ________
Other ________

3

You receive	50	54	59	63	68	72	76	81	85
Other receives	100	98	96	94	93	91	89	87	85

You ________
Other ________

4

You receive	50	54	59	63	68	72	76	81	85
Other receives	100	89	79	68	58	47	36	26	15

You ________
Other ________

5

You receive	100	94	88	81	75	69	63	56	50
Other receives	50	56	63	69	75	81	88	94	100

You ________
Other ________

6

You receive	100	98	96	94	93	91	89	87	85
Other receives	50	54	59	63	68	72	76	81	85

You ________
Other ________

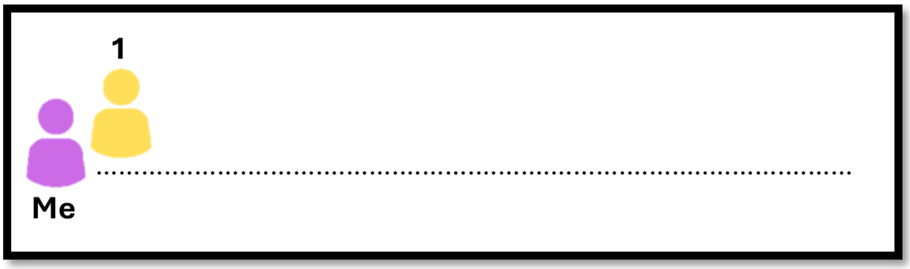

In this round you will be making a series of decisions about allocating resources between you and the person you chose for **position 1**.

Please write the initials of this person here: ______________________

Each arrow pointing to the right indicates one question. For each question, please indicate the distribution you prefer most by marking the respective position along the midline. You can only make one mark for each question.

Your decisions will yield money for both you and the person at **position 1**. There are no right or wrong answers, this is all about personal preferences. After you have made your decision, write the resulting distribution of money on the spaces on the right.

1

You receive	85	85	85	85	85	85	85	85	85
Other receives	85	76	68	59	50	41	33	24	15

You ________

Other ________

2

You receive	85	87	89	91	93	94	96	98	100
Other receives	15	19	24	28	33	37	41	46	50

You ________

Other ________

3

You receive	50	54	59	63	68	72	76	81	85
Other receives	100	98	96	94	93	91	89	87	85

You ________

Other ________

4

You receive	50	54	59	63	68	72	76	81	85
Other receives	100	89	79	68	58	47	36	26	15

You ________

Other ________

5

You receive	100	94	88	81	75	69	63	56	50
Other receives	50	56	63	69	75	81	88	94	100

You ________

Other ________

6

You receive	100	98	96	94	93	91	89	87	85
Other receives	50	54	59	63	68	72	76	81	85

You ________

Other ________

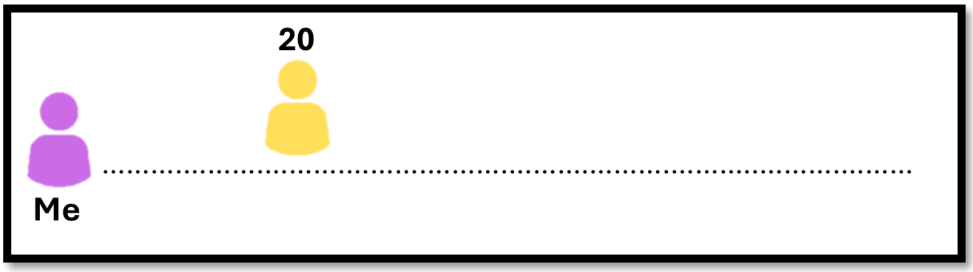

In this round you will be making a series of decisions about allocating resources between you and the person you chose for **position 20**.

Please write the initials of this person here: ______________________

Each arrow pointing to the right indicates one question. For each question, please indicate the distribution you prefer most by marking the respective position along the midline. You can only make one mark for each question.

Your decisions will yield money for both you and the person at **position 20**. There are no right or wrong answers, this is all about personal preferences. After you have made your decision, write the resulting distribution of money on the spaces on the right.

1

You receive	85	85	85	85	85	85	85	85	85
Other receives	85	76	68	59	50	41	33	24	15

You ________
Other ________

2

You receive	85	87	89	91	93	94	96	98	100
Other receives	15	19	24	28	33	37	41	46	50

You ________
Other ________

3

You receive	50	54	59	63	68	72	76	81	85
Other receives	100	98	96	94	93	91	89	87	85

You ________
Other ________

4

You receive	50	54	59	63	68	72	76	81	85
Other receives	100	89	79	68	58	47	36	26	15

You ________
Other ________

5

You receive	100	94	88	81	75	69	63	56	50
Other receives	50	56	63	69	75	81	88	94	100

You ________
Other ________

6

You receive	100	98	96	94	93	91	89	87	85
Other receives	50	54	59	63	68	72	76	81	85

You ________
Other ________

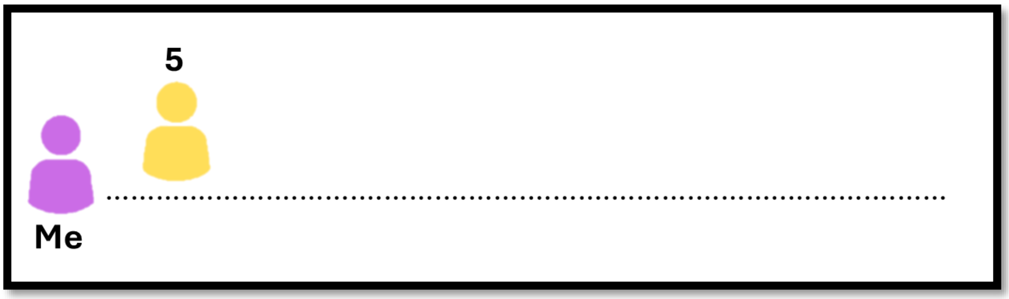

In this round you will be making a series of decisions about allocating resources between you and the person you chose for **position 5**.

Please write the initials of this person here: ______________________

Each arrow pointing to the right indicates one question. For each question, please indicate the distribution you prefer most by marking the respective position along the midline. You can only make one mark for each question.

Your decisions will yield money for both you and the person at **position 5**. There are no right or wrong answers, this is all about personal preferences. After you have made your decision, write the resulting distribution of money on the spaces on the right.

1

You receive	85	85	85	85	85	85	85	85	85
Other receives	85	76	68	59	50	41	33	24	15

You ________
Other ________

2

You receive	85	87	89	91	93	94	96	98	100
Other receives	15	19	24	28	33	37	41	46	50

You ________
Other ________

3

You receive	50	54	59	63	68	72	76	81	85
Other receives	100	98	96	94	93	91	89	87	85

You ________
Other ________

4

You receive	50	54	59	63	68	72	76	81	85
Other receives	100	89	79	68	58	47	36	26	15

You ________
Other ________

5

You receive	100	94	88	81	75	69	63	56	50
Other receives	50	56	63	69	75	81	88	94	100

You ________
Other ________

6

You receive	100	98	96	94	93	91	89	87	85
Other receives	50	54	59	63	68	72	76	81	85

You ________
Other ________

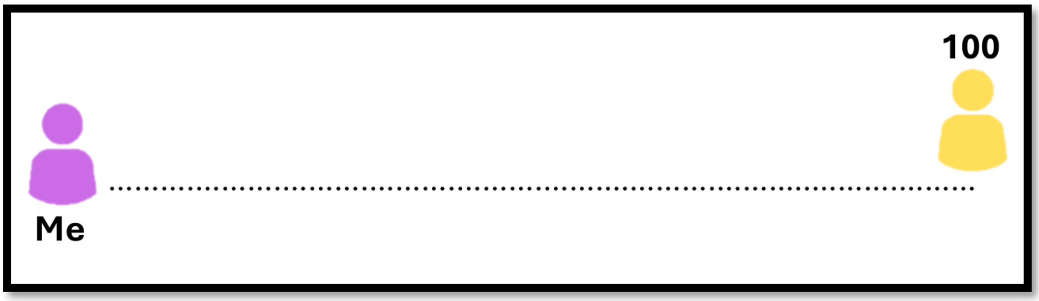

In this round you will be making a series of decisions about allocating resources between you and the person you chose for **position 100**.

This is a stranger who you have never seen before.

Each arrow pointing to the right indicates one question. For each question, please indicate the distribution you prefer most by marking the respective position along the midline. You can only make one mark for each question.

Your decisions will yield money for both you and the person at **position 100**. There are no right or wrong answers, this is all about personal preferences. After you have made your decision, write the resulting distribution of money on the spaces on the right.

1

You receive	85	85	85	85	85	85	85	85	85
Other receives	85	76	68	59	50	41	33	24	15

You ________
Other ________

2

You receive	85	87	89	91	93	94	96	98	100
Other receives	15	19	24	28	33	37	41	46	50

You ________
Other ________

3

You receive	50	54	59	63	68	72	76	81	85
Other receives	100	98	96	94	93	91	89	87	85

You ________
Other ________

4

You receive	50	54	59	63	68	72	76	81	85
Other receives	100	89	79	68	58	47	36	26	15

You ________
Other ________

5

You receive	100	94	88	81	75	69	63	56	50
Other receives	50	56	63	69	75	81	88	94	100

You ________
Other ________

6

You receive	100	98	96	94	93	91	89	87	85
Other receives	50	54	59	63	68	72	76	81	85

You ________
Other ________

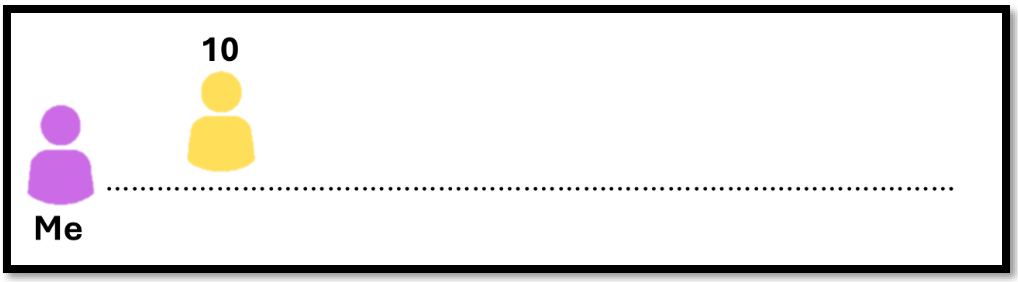

In this round you will be making a series of decisions about allocating resources between you and the person you chose for **position 10**.

Please write the initials of this person here: ________________________

Each arrow pointing to the right indicates one question. For each question, please indicate the distribution you prefer most by marking the respective position along the midline. You can only make one mark for each question.

Your decisions will yield money for both you and the person at **position 10**. There are no right or wrong answers, this is all about personal preferences. After you have made your decision, write the resulting distribution of money on the spaces on the right.

1

You receive	85	85	85	85	85	85	85	85	85
Other receives	85	76	68	59	50	41	33	24	15

You ________
Other ________

2

You receive	85	87	89	91	93	94	96	98	100
Other receives	15	19	24	28	33	37	41	46	50

You ________
Other ________

3

You receive	50	54	59	63	68	72	76	81	85
Other receives	100	98	96	94	93	91	89	87	85

You ________
Other ________

4

You receive	50	54	59	63	68	72	76	81	85
Other receives	100	89	79	68	58	47	36	26	15

You ________
Other ________

5

You receive	100	94	88	81	75	69	63	56	50
Other receives	50	56	63	69	75	81	88	94	100

You ________
Other ________

6

You receive	100	98	96	94	93	91	89	87	85
Other receives	50	54	59	63	68	72	76	81	85

You ________
Other ________

This is the end of the task. Thank you for taking part!

If you have any comments concerning the task, you can write them here.

You can also leave this section blank.
